# The Effect of OnabotulinumtoxinA on Headache Intensity and Number of Monthly Headache Days in Individuals with Chronic Migraine with Different Levels of Neck Disability

**DOI:** 10.3390/toxins15120685

**Published:** 2023-12-06

**Authors:** Dilara Onan, Halime Arıkan, Paolo Martelletti

**Affiliations:** 1 Department of Physiotherapy and Rehabilitation, Faculty of Heath Sciences, Yozgat Bozok University, Yozgat 66100, Turkey; dilaraonan@gmail.com; 2 Department of Physiotherapy and Rehabilitation, Faculty of Health Sciences, Tokat Gaziosmanpasa University, Tokat 60000, Turkey; halimearikan92@gmail.com; 3 School of Health Sciences, Unitelma Sapienza University, 00161 Rome, Italy

**Keywords:** chronic migraine, neck pain, neck disability, pain intensity, number of monthly headache days, OnabotulinumtoxinA, quality-of-life

## Abstract

One of the treatment methods used in chronic migraine is OnabotulinumtoxinA. The effects of OnabotulinumtoxinA on headache intensity (HI) and number of monthly headache days (NMHD) in chronic migraine (CM) patients classified according to neck disability levels are unknown. Our aim was to investigate the effect of OnabotulinumtoxinA on the HI and the NMHD in individuals with CM with different levels of neck disability. One hundred sixteen patients were enrolled in the study. The OnabotulinumtoxinA protocol was administered as per Follow-the-Pain PREEMPT. The Neck Disability Index was used to evaluate neck disability. Primary outcome measures were headache intensity, assessed with the Visual Analogue Scale, and the number of monthly headache days recorded from patients’ diaries. Secondary outcome measures were migraine disability, assessed with the Migraine Disability Assessment Test, and quality-of-life, assessed with the Headache Impact Test-6. All assessments were made at baseline and end of the treatment. The OnabotulinumtoxinA treatment showed a greater improvement effect in the number of monthly headache days (*p* = 0.000) and migraine disability (*p* = 0.000) parameters in the severe and complete disability groups. CM patients with complete and severe neck disability received the most benefit in reducing the NMHD at 3 months after OnabotulinumtoxinA treatment, but the HI decreased at a similar level in all neck disability groups.

## 1. Introduction

In chronic migraine (CM), which is among the primary headaches [1], secondary pain complaints such as neck pain (NP) that patients experience together with headaches have attracted the attention of researchers in recent years [2,3,4,5,6,7,8,9,10,11,12]. It is a matter of increasing curiosity among researchers as the trigeminal and cervical afferents at the trigeminocervical junction combine to perceive pain in the head and neck regions, and the process is addressed not only in the head region but also in the neck region [2,3].

Nociceptive stimuli from the dura mater and cervical region, when combined with secondary neurons in the trigeminocervical complex, cause a series of activations as a result of long-term stimulation of the caudal nucleus of the trigeminal nerve. This activation is considered an important point in the relationship between migraine and neck pain [13,14]. Continuing the activation for a long time can make the condition chronic and cause neck disorders such as pain or disability to become more severe [13,14,15]. The results of a cross-sectional study reported that NP was twice as common in patients with CM patients compared to episodic migraine [15]. When the parameters in the Neck Disability Index (NDI), which assesses neck disability and includes postural adjustments in daily life [16], are examined, the neck disability level increases as the impairment in activities such as driving, personal care, weight lifting, or reading increases [15]. In literature studies, the reasons for neck disability for CM have been questioned, and it has been stated that the cervical spine has less mobility without any difference in muscle activity, and craniocervical test endurance performance and neck movement speed in activities are lower [17,18,19]. According to the results of a hospital-based clinical study, an average 2 cm increase in headache intensity in CM patients with mild neck disability in the NDI could result in complete neck disability. In CM patients with moderate neck disability levels, it has been found that an average 1 cm increase in headache intensity can cause complete neck disability [20]. Therefore, evaluating patients in clinics in terms of neck disability may shed light on how much changes in headache intensity may affect neck disability.

OnabotulinumtoxinA is a drug with proven effectiveness used in the preventive treatment of CM by inhibiting glutamate A and P substances and preventing sensitivity with the calcitonin gene-related peptide secreted from active sensory nerves [21,22,23]. OnabotulinumtoxinA is recommended by the European Headache Federation [24]. According to the usage algorithm, onabotulinumtoxinA application is injected into a total of 31–39 areas (155 U–195 U for head and neck muscles), with 5 U in each area, every 12 weeks [25,26]. In a clinical study using this algorithm, neck and headache intensity, neck disability, and migraine disability decreased significantly in the third month after OnabotulinumtoxinA application in CM patients. In addition, the number of monthly headache days decreased from 20 to 6 [27]. Investigating the effects of the methods used in the treatment of CM on NP or disability, the intensity of headache, and the number of headache days per month will be useful for clinicians and researchers in predicting the patient’s condition.

To our knowledge, there is no study investigating the effect of OnabotulinumtoxinA treatment on headache intensity and number of monthly headache days in patients with CM classified according to neck disability. Therefore, in this study, we aim to contribute to the literature to elucidate the relationship between CM and NP by investigating the effect of OnabotulinumtoxinA on the intensity of headache and the number of headache days in individuals with CM with different levels of neck disability.

## 2. Results

A total of 116 individuals, 106 women (91.38%) and 10 men (8.62%), were included in the study. Individuals were grouped as mild, moderate, severe, and complete disability according to their neck disability levels. The number of individuals in these groups was 15, 22, 44, and 35, respectively. All groups were similar in terms of demographic characteristics (*p* < 0.05) (Table 1).

The intensity of headache, number of monthly headache days, migraine disability, and quality of life showed significant improvement in each group after the OnabotulinumtoxinA treatment [*p* = 0.000]. As a result of intergroup comparisons, a difference was seen in headache intensity as the primary outcome measure, migraine disability and quality of life as the secondary outcome measures before the OnabotulinumtoxinA treatment, and in the number of monthly headache days as the primary outcome measure after the OnabotulinumtoxinA intervention. Groups with differences were highlighted in Table 2.

It was examined which group showed a greater healing effect after OnabotulinumtoxinA intervention with the evaluated parameters. In this examination, the improvement in the intensity of headache and quality of life was similar between groups. The groups had different extents of improvement in the number of monthly headache days and migraine disability. OnabotulinumtoxinA intervention had a greater improvement effect in these parameters in the severe and complete disability groups (Table 3).

## 3. Discussion

This retrospective study aimed to investigate the effect of OnabotulinumtoxinA, used in the preventive treatment of CM, on headache intensity and the number of headache days in individuals with CM with different neck disability levels. Our hypothesis was partially confirmed. In the third-month results after OnabotulinumtoxinA application, when the patients were divided into groups according to their neck disability levels, the greatest improvement in the number of monthly headache days, which is one of the primary outcome measures, was in the complete and severe disability groups, respectively. However, headache intensity decreased to a similar level in almost all neck disability groups. While the greatest improvement in migraine disability, which is one of the secondary outcome measures, was found to be significant in the complete and severe disability groups, respectively, there was no difference between the groups in terms of improvement in the quality of life.

Neck disability is known as a parameter that evaluates posture-related impairments in daily activities in patients with NP in clinics. Neck pain and disability, which are among the complaints of migraine patients in the pain process in which the trigeminocervical junction plays a role, is a situation that should be questioned by physicians and physiotherapists, especially in the chronic process, and the treatment and rehabilitation process should be addressed quickly. The NDI, which is the golden evaluation parameter of a neck disability, has items that evaluate neck and headache intensity impairment in daily postural activities such as reading, lifting, working, and driving [16]. Considering the parameters included in the NDI, it is recommended to evaluate migraine patients with neck pain to determine their disability in daily living activities [28]. Florencio et al.’s study determined that neck disability was associated with migraine attacks. The fact that the parameters in which migraine patients experience the most disability in the NDI are headache, reading, and lifting activities, respectively, reveals that as the intensity of the attacks increases, the intensity of neck problems and, therefore, the deterioration in daily postural activities increases [15]. Di Antonio et al. showed that in migraine patients with NP, the cervical active range of motion was less than in migraine patients without neck pain, the headache frequency was higher, the disability was worse, and the pain threshold in the cervical region was also lower [29]. In the previous clinical study, it was stated that an average of 2 cm increase in the intensity of headache at the mild neck disability level progressed to the complete neck disability, while an average 1 cm increase in the intensity of headache at the moderate neck disability level also progressed to the complete neck disability level [20]. Therefore, based on the results of clinical studies, when migraine attacks are considered, it can be understood that the increase in the headache frequency, the intensity of headaches, and the number of monthly painful days can negatively affect neck disability. At this point, we think it is important to comprehensively evaluate the necks of patients who have CM and present to clinics with neck problems and to include neck-related problems in the treatment. Among the reasons for this important point is that the trigeminocervical junction plays a role in the coexistence of NP and headache. The causes thought to be effective in pathophysiology are peripheral sensitization and central sensitization. It is suggested that a stimulus that may cause pain and disability in the cervical musculoskeletal system in peripheral sensitization may increase the frequency of migraine by increasing the sensitivity of the trigeminocervical junction [18,30,31,32]. In central sensitization, increased migraine symptoms may increase the sensitivity and repetitive excitability of neurons in the trigeminocervical junction, causing a situation that may result in hyperalgesia and neck pain/disability [15,28,33]. In addition, the high number of headache days and the frequency of attacks and the short time between attacks may make it difficult to answer the question of whether it is a cause or an effect in terms of neck pain/disability [29]. Therefore, at this point, finding out which one is the source of pain arouses the curiosity of researchers. The evaluation of the patients’ symptoms before, during, and after the attack, both by themselves and by the researchers, can provide valuable and important results.

Secondly, individuals’ hypersensitivity to pain and secondary problems may cause them to use their head and neck regions less in daily living activities, and cervical spine stability may weaken. Compared to migraine patients without NP and healthy controls, migraine patients with NP have a less active cervical joint range of motion, lower pain threshold in the cervical region, and increased sensitivity in the cervical region muscles that may cause individuals to need to protect themselves from pain. Therefore, further reducing the movement of the cervical region and its use in daily living activities may be a reason for the decrease in cervical region muscle strength and stability. This may increase the severity of the disability in daily living activities [15].

The treatment process of CM is handled pharmacologically and non-pharmacologically [34]. OnabotulinumtoxinA treatment is an effective and well-tolerated treatment that plays a role in pain inhibition in chronic migraine by inhibiting neurotransmitters, substance P, calcitonin-related peptide, and glutamate A in the sensory pathways of the trigeminal nerve. Considering the neck areas where OnabotulinumtoxinA treatment is applied, it is seen that it provides improvements in neck pain and disability, which are complaints of chronic migraine patients. In a hospital-based study, OnabotulinumtoxinA treatment was found to be effective for 3 months in reducing the intensity of NP, neck disability, intensity of headache, and number of monthly headache days and increasing the quality of life associated with CM [27]. The results of a systematic review reported that OnabotulinumtoxinA treatment in migraine was effective in reducing disability, headache intensity, frequency, and number of monthly headache days [35]. In a study involving multiple European headache centers, it was stated that the rate of excellent responders to OnabotulinumtoxinA treatment in terms of frequency at the end of the third month was 3.9% (*n* = 112/2879), and this rate was predictive of improvement in the sixth and ninth months [36]. To our knowledge, our study is the first in the literature to separate CM patients according to their neck disability levels, apply OnabotulinumtoxinA treatment, and investigate migraine symptoms. In the 3-month results of our study, the greatest improvement in the reduction in the number of monthly headache days was significant in the complete (−14.69 ± 7.87 days) and severe (−12.20 ± 8.92 days) disability groups, respectively. However, the level of reduction in headache intensity was similar in almost all neck disability groups. While the greatest improvement in migraine disability was significant in the complete (−42.20 ± 42.11) and severe (−31.32 ± 29.63) disability groups, respectively, the improvement in quality of life was similar between the groups. Therefore, our hypothesis was partially confirmed by finding a significant improvement in the number of headache days among the number of headache days and headache intensity parameters. The groups with the highest improvements in the number of headache days and the MIDAS were complete disability and severe disability, and there was no difference between these two groups in terms of these parameters. On the other hand, it was expected that the improvement would be greater in the groups where the neck disability was complete and severe compared to the groups where the neck disability was mild and moderate. From these results, we can say that neck disability was at the level of complete and severe disability, and these two groups were not superior to each other in terms of benefiting from the treatment, increasing the benefit seen from OnabotulinumtoxinA treatment. The condition of patients with mild and moderate neck disabilities may not cause enough complaints to bring the patients to the clinic. In order for a neck disability to benefit from treatment, clinicians should pay attention to preventing it from transitioning from moderate to severe. Therefore, this finding may be an important marker for detecting neck disability in chronic migraine patients at early levels before it progresses.

In our results, there was no change in the intensity of headache between the neck disability groups after OnabotulinumtoxinA treatment, but there was a significant improvement in the number of monthly headache days, suggesting that the neck disability may reflect the number of painful days the patients had in a month. Because headache intensity is a subjective experience, patients may feel their headache intensity by rating it very high during attacks, regardless of whether the number of headache days increases or decreases. Therefore, the decrease in the number of headache days may contribute to the improvement of neck disability by allowing more use of the head and neck in daily life. Neck disability also seems to progress in parallel with general migraine disability, with the MIDAS results showing that the improvement in complete and severe neck disability levels was greatest. No difference was found between the groups in quality of life. In addition, although studies investigating OnabotulinumtoxinA treatment and neck pain/disability-related problems in chronic migraine patients are limited in the literature, physiotherapy and rehabilitation approaches applied together with OnabotulinumtoxinA treatment may increase the effectiveness of improvements in migraine symptoms and neck problems [37]. The results of a 3-month observational cohort study are very positive. In CM patients, it has been shown that headache intensity and frequency decreased only in the group where OnabotulinumtoxinA treatment was applied and in the group where OnabotulinumtoxinA treatment and physiotherapy and rehabilitation approaches (manual therapy and active exercises) were applied together. However, it was stated that the improvement was greater in the group where both approaches were applied [38]. Therefore, despite the limited studies in the literature, it is thought that OnabotulinumtoxinA treatment provides improvements in migraine symptoms and neck complaints in chronic migraine patients and may increase the recovery effectiveness of treatment programs that can be supported by physical therapy and rehabilitation approaches.

Our study has some limitations. Since our study is retrospective, it is normal for there to be differences in the number of patients in the groups. Accordingly, there may be differences in the parameters evaluated before treatment. However, taking this into consideration, we compared the intra-group changes of the parameters after the treatment in order to accurately evaluate the treatment effectiveness. Again, since our study is a retrospective study, there are no long-term follow-up results. Therefore, the effectiveness of the treatment was limited to 3-month results. As another limitation, neck disability was evaluated only by questionnaire. Although the NDI is a good marker for assessing neck disability, we think that the lack of objective test evaluations from daily living activities is another limitation of our study.

In addition to the NDI in the evaluation of neck disability, evaluating the condition of chronic migraine patients in their daily living activities with tests may provide more objective results. On the other hand, while pharmacological treatments continue in chronic migraine, we think it is important to include non-pharmacological approaches in the process. Physiotherapy and rehabilitation approaches are used as manual and exercise practices in chronic migraine patients [39,40,41,42,43,44]. Physiotherapy and rehabilitation approaches applied in addition to OnabotulinumtoxinA application or drug treatments may provide benefits that will increase the quality of life, especially when exercises are transformed into routine programs that can reduce patients’ pharmacological dependence.

## 4. Conclusions

Chronic migraine patients often experience neck pain and disability problems originating from the trigeminocervical junction. Although the evaluation and treatment process is generally aimed at chronic migraine, it is important to also address neck problems. According to our results, patients with complete and severe neck disability received the most benefit in reducing the number of monthly headache days at the end of the third month of OnabotulinumtoxinA treatment, but the intensity of pain decreased at a similar level in all neck disability groups. Since pain intensity is a subjective experience, neck disability may be affected by the number of days spent with a headache per month rather than the intensity of the headache felt during the attack. Migraine disability improved the most in the groups with complete and severe neck disability. It has been observed that patients with high neck disability also experience severe migraine disability and give positive results with treatment. Our results contribute to the research field on chronic migraine and neck complaints in the literature. We think that in future studies, in addition to long-term follow-up and OnabotulinumtoxinA treatment, randomized controlled studies designed with non-pharmacological methods such as physiotherapy and rehabilitation approaches should investigate the effects of migraine symptoms and neck complaints.

The outcomes of this study can be read in the light of an overall therapeutic offer to individuals with chronic migraine in order to guarantee flexible and sustainable medical and pharmacological care for all in different global geographic areas [33,34].

## 5. Materials and Methods

The ethical permission of this retrospective, open-label, real-world study was received from Tokat Gaziosmanpaşa University, Faculty of Medicine Clinical Research Ethics Committee (23-KAEK-228, the date of permission: 28 September 2023) to conduct this research.

### 5.1. Participant Selection

The research included individuals aged 18–65 who applied to the Headache Clinic of Sant’Andrea Hospital of Sapienza University of Rome between 1 June 2022 and 4 November 2022. One hundred sixteen individuals diagnosed with CM, according to the *International Classification of Headache Disorders, 3rd Edition*, by an internist who specializes in headaches were included.

Individuals who were diagnosed with CM patients with headaches that occur more than 15 days a month for more than three months and have migraine headaches on at least eight days of the month, who are between the ages of 18–65, and who experience neck pain during migraine attacks were included in the study.

Individuals with any headache diagnosis other than CM, cervical pathology/diagnosis that causes neck pain, and a history of any systemic disease such as malignant, inflammatory, acute fracture, surgical, neurological, psychological, or rheumatological were excluded from the research. Chronic migraineurs were also excluded and given additional treatments prior to botox or any prophylactics.

### 5.2. Outcome Measures

Demographic information of all patients was recorded. Patients were divided according to their NDI levels. First, the primary outcome measures, the intensity of headache, and number of monthly headache days were questioned. Then, the secondary outcome measures, the Migraine Disability Assessment (MIDAS) and the Headache Impact Test (HIT-6) were evaluated. All outcome measures were recorded at baseline and three months after OnabotulinumtoxinA treatment.

### 5.3. The Primary Outcome Measures

#### 5.3.1. Visual Analog Scale

The intensity of headache was evaluated with the Visual Analog Scale (VAS). A horizontal line was drawn with a 10 cm long ruler for VAS. The beginning of the line was expressed to individuals as 0: “I have no pain”. Moreover, the end of the line was expressed to individuals as 10: “I have unbearable pain”. Individuals were asked to mark their headache intensity between these two points. The distance from the determined point to the starting point was measured, and this distance was recorded as headache intensity [45,46].

#### 5.3.2. Number of Monthly Headache Days

The number of monthly headache days was noted from the headache diaries given to the CM patients.

#### 5.3.3. Neck Disability Index

The neck disability of the individuals was evaluated with the Neck Disability Index (NDI) [16]. The NDI consists of 10 questions in total. The total score is between 0 and 50. It is considered as 0–4 points: “no disability”, 5–14 points: “mild disability”, 15–24 points: “moderate disability”, 25–35 points: “severe disability”, and 35 points and above: “complete disability”. The NDI evaluates head and neck pain, self-care, concentration, work, driving, and sleep. The Italian version, validity, and reliability study of the NDI was conducted by Monticone et al. [47].

### 5.4. The Secondary Outcome Measures

#### 5.4.1. Migraine Disability Assessment Test

The impact of migraine on individuals was evaluated with the Migraine Disability Assessment Test (MIDAS) [48,49]. It evaluates the disability of the last 3 months. The test consists of 5-item self-administered items that include activities at work/school, housework, family, and social or leisure time. The total number of days missed in these activities is the total score. The disability levels are minimal (0–5 points), mild (6–10 points), moderate (11–20 points), or severe (≥21 points). The Italian version of the questionnaire, its validity, and reliability studies were conducted by D’amico et al. [50].

#### 5.4.2. Headache Impact Test-6

The Headache Impact Test-6 (HIT-6) is a quality-of-life questionnaire that evaluates vitality, pain, psychological condition, sociability, role, and cognitive functioning and includes six items [51,52]. The total score ranges from 36 to 78 points (0–49: little/no effect, 50–55: some effect, 56–59: substantial effect, and 60–78: severe effect). As the score increases, it indicates that the quality of life worsens. The validity and reliability study of the Italian version of the questionnaire was conducted by Gandek et al. [53].

### 5.5. Intervention

The OnabotulinumtoxinA was administered to individuals by an internal medicine specialist with headache expertise. A total of 39 regions were included in the application, including glabellar, frontal, temporal, occipital regions, upper cervical paraspinal, and trapezius muscles. OnabotulinumtoxinA application was completed by injecting 195 U, 5 units, into each region according to the PREEMPT protocol as Follow-the-Pain [25,26]. Individuals were evaluated regarding the intensity of headache, number of headache days, neck disability, migraine disability, and quality-of-life before receiving OnabotulinumtoxinA treatment and 3 months after the application.

### 5.6. Sample Size

Data from individuals’ routine evaluations in the clinic were used on the effects of 3-month application after OnabotulinumtoxinA treatment. Power analysis was performed with the G*Power 3.1.9.4 program to determine the number of individuals included in the study. Headache intensity via the NDI was considered the primary outcome measurement variable. Taking the effect size as 0.34 and alpha as 0.05, the number of individuals required for the study was at least 68. The power of the study was determined as 98% with 68 individuals.

### 5.7. Statistical Analysis

Analyses were performed with SPSS (Statistical Package for the Social Sciences) version 22. Descriptive statistics were given as frequency, mean, and standard deviation. If the variables were normally distributed in intra-group comparisons, Repeated Measures Analysis of Variance was used in multi-group comparisons, and if the variables were not normally distributed, Friedman Analysis was used in multi-group comparisons. In comparisons between groups, One-Way Analysis of Variance was used if the variables were normally distributed, and Kruskal–Wallis Analysis was used if the variables were not normally distributed. As a result of differences between groups, Bonferroni correction was used for parametric data, while the Mann–Whitney U Test was used for nonparametric data in intergroup comparisons. In statistical analysis, *p* < 0.05 was considered significant.

## Figures and Tables

**Table 1:** Demographic characteristics.

	Mild (*n* = 15)	Moderate (*n* = 22)	Severe (*n* = 44)	Complete (*n* = 35)	*p*
**Age (years)**	48.20 ± 11.48	48.09 ± 12.00	52.23 ± 9.38	50.43 ± 8.98	0.384 ^β^
**Weight (kg)**	62.93 ± 12.94	63.77 ± 11.93	65.36 ± 11.37	67.00 ± 13.97	0.690 ^β^
**Height (cm)**	166.07 ± 8.12	166.27 ± 6.24	164.14 ± 6.79	163.74 ± 7.03	0.450 ^α^
**BMI (kg/m^2^)**	22.79 ± 4.17	23.04 ± 3.94	24.23 ± 3.77	24.93 ± 4.36	0.211 ^β^
**Migraine Diagnosis Time (years)**	17.67 ± 12.62	22.82 ± 13.54	22.23 ± 14.89	22.94 ± 14.64	0.683 ^β^
**Gender** ***Female*** ***Male***	13 [86.7%] 2 [13.3%]	20 [90.9%] 2 [9.1%]	42 [95.5%] 2 [4.5%]	31 [88.6%] 4 [11.4%]	-

Kg: Kilogram; cm: Centimeter; BMI: Body Mass Index; ^α^: One-Way Analysis of Variance; ^β^: Kruskal–Wallis Analysis.

**Table 2:** Intra- and inter-group effects of OnabotulinumtoxinA intervention.

The Primary Outcome Measures	Mild (*n* = 15)	Moderate (*n* = 22)	Severe (*n* = 44)	Complete (*n* = 35)	*p*	Posthoc
***Number of monthly headache days (B.O.)***	15.73 ± 9.17	18.68 ± 8.65	20.36 ± 9.40	20.60 ± 7.79	0.273 ^β^	
***Number of monthly headache days (A.O.)***	6.93 ± 5.26	13.41 ± 9.41	8.16 ± 7.48	5.91 ± 4.66	**0.007** ^β^	Moderate > Mild (*p* = 0.035 **^λ^**) Moderate > Severe (*p* = 0.015 **^λ^**) Moderate > Complete (*p* = 0.001 **^λ^**)
***p***	**0.001 ^§^**	**0.001 ^§^**	**0.000 ^§^**	**0.000 ^§^**		
***VAS (B.O.)***	7.87 ± 1.36	8.64 ± 1.14	9.32 ± 0.93	9.60 ± 1.03	**0.000** ^β^	Severe > Mild (*p* = 0.000 **^λ^**) Complete > Mild (*p* = 0.000 **^λ^**) Severe > Moderate (*p* = 0.015 **^λ^**) Complete > Moderate (*p* = 0.000 **^λ^**) Complete > Severe (*p* = 0.032 **^λ^**)
***VAS (A.O.)***	4.40 ± 2.23	4.73 ± 1.98	5.41 ± 2.14	4.83 ± 2.26	0.385 ^β^	
***p***	**0.001 ^§^**	**0.000 ^§^**	**0.000 ^§^**	**0.000 ^§^**		
**The Secondary Outcome Measures**	**Mild** **(*n* = 15)**	**Moderate** **(*n* = 22)**	**Severe** **(*n* = 44)**	**Complete** **(*n* = 35)**	***p***	**Posthoc**
***MIDAS total (B.O.)***	26.40 ± 25.65	28.73 ± 17.89	58.98 ± 61.71	71.69 ± 63.78	**0.000** ^β^	Severe > Mild (*p* = 0.010 **^λ^**) Complete > Mild (*p* = 0.000 **^λ^**) Severe > Moderate (*p* = 0.024 **^λ^**) Complete > Moderate (*p* = 0.000 **^λ^**)
***MIDAS total (A.O.)***	11.40 ± 12.33	15.23 ± 15.64	27.66 ± 45.62	29.49 ± 37.01	0.123 ^β^	
***p***	**0.001 ^§^**	**0.000 ^§^**	**0.000 ^§^**	**0.000 ^§^**		
***HIT-6 (B.O.)***	63.60 ± 8.14	65.27 ± 5.41	69.59 ± 5.43	71.71 ± 5.11	**0.000** ^α^	Severe > Mild (*p* = 0.009 ^£^) Complete > Mild (*p* = 0.001 ^£^) Severe > Moderate (*p* = 0.005 ^£^) Complete > Moderate (*p* = 0.000 ^£^)
***HIT-6 (A.O.)***	53.87 ± 7.73	56.45 ± 7.91	58.52 ± 8.31	57.51 ± 7.51	0.254 ^α^	
***p***	**0.000 ^µ^**	**0.000 ^µ^**	**0.000 ^µ^**	**0.000 ^µ^**		

B.O.: Before OnabotulinumtoxinA; A.O.: After OnabotulinumtoxinA; VAS: Visual Analog Scale; MIDAS: Migraine Disability Assessment Test; HIT-6: Headache Impact Test-6; ^§^: Wilcoxon Test; ^µ^: Paired Samples *t* Test; ^α^: One-Way Analysis of Variance; ^β^: Kruskal–Wallis Analysis; ^λ^: Mann–Whitney U Test; ^£^: Bonferroni Statistics.

**Table 3:** Differences caused by OnabotulinumtoxinA intervention in parameters according to neck disability level.

The Primary Outcome Measures	Mild (*n* = 15)	Moderate (*n* = 22)	Severe (*n* = 44)	Complete (*n* = 35)	*p*
***Number of headache days***	−8.80 ± 7.65	−5.27 ± 7.72	−12.20 ± 8.92	−14.69 ± 7.87	**0.000 ^β^**
**Posthoc**	Mild > Moderate (***p* = 0.029 ^λ^**); Complete > Mild (***p* = 0.012 ^λ^**); Severe > Moderate (***p* = 0.002 ^λ^**); Complete > Moderate (***p* = 0.000 ^λ^**)				
***VAS***	−3.47 ± 1.81	−3.91 ± 2.04	−3.91 ± 2.27	−4.77 ± 2.33	0.354 ^β^

**The Secondary Outcome Measures**	**Mild** **(*n* = 15)**	**Moderate** **(*n* = 22)**	**Severe** **(*n* = 44)**	**Complete** **(*n* = 35)**	***p***
***MIDAS***	−15.00 ± 17.22	−13.50 ± 15.41	−31.32 ± 29.63	−42.20 ± 42.11	**0.000 ^β^**
**Posthoc**	Severe > Mild (***p* = 0.021 ^λ^**); Complete > Mild (***p* = 0.001 ^λ^**); Severe > Moderate (***p* = 0.003 ^λ^**); Complete > Moderate (***p* = 0.000 ^λ^**)				
***HIT-6***	−9.73 ± 7.41	−8.82 ± 6.92	−11.07 ± 8.00	−14.20 ± 9.31	0.123 ^β^

VAS: Visual Analog Scale; MIDAS: Migraine Disability Assessment Test; HIT-6: Headache Impact Test-6; ^β^: Kruskal–Wallis Analysis; ^λ^: Mann–Whitney U Test.

## Data Availability

The data presented in this study are available in this article.
